# Coactivation pattern analysis reveals altered salience network dynamics in children with autism spectrum disorder

**DOI:** 10.1162/netn_a_00163

**Published:** 2020-12-01

**Authors:** Emily Marshall, Jason S. Nomi, Bryce Dirks, Celia Romero, Lauren Kupis, Catie Chang, Lucina Q. Uddin

**Affiliations:** Department of Psychology, University of Miami, Coral Gables, FL, USA; Department of Psychology, University of Miami, Coral Gables, FL, USA; Department of Psychology, University of Miami, Coral Gables, FL, USA; Department of Psychology, University of Miami, Coral Gables, FL, USA; Department of Psychology, University of Miami, Coral Gables, FL, USA; Department of Electrical Engineering and Computer Science, Vanderbilt University, Nashville, TN, USA; Department of Biomedical Engineering, Vanderbilt University, Nashville, TN, USA; Vanderbilt University Institute of Imaging Science, Vanderbilt University, Nashville, TN, USA; Department of Psychology, University of Miami, Coral Gables, FL, USA; Neuroscience Program, University of Miami Miller School of Medicine, Miami, FL, USA

**Keywords:** Anterior insula, Dynamic coactivation patterns, Functional connectivity, Lateral fronto-parietal network, Medial fronto-parietal network, Midcingulo-insular network

## Abstract

Brain connectivity studies of autism spectrum disorder (ASD) have historically relied on static measures of functional connectivity. Recent work has focused on identifying transient configurations of brain activity, yet several open questions remain regarding the nature of specific brain network dynamics in ASD. We used a dynamic coactivation pattern (CAP) approach to investigate the salience/midcingulo-insular (M-CIN) network, a locus of dysfunction in ASD, in a large multisite resting-state fMRI dataset collected from 172 children (ages 6–13 years; *n* = 75 ASD; *n* = 138 male). Following brain parcellation by using independent component analysis, dynamic CAP analyses were conducted and *k*-means clustering was used to determine transient activation patterns of the M-CIN. The frequency of occurrence of different dynamic CAP brain states was then compared between children with ASD and typically developing (TD) children. Dynamic brain configurations characterized by coactivation of the M-CIN with central executive/lateral fronto-parietal and default mode/medial fronto-parietal networks appeared less frequently in children with ASD compared with TD children. This study highlights the utility of time-varying approaches for studying altered M-CIN function in prevalent neurodevelopmental disorders. We speculate that altered M-CIN dynamics in ASD may underlie the inflexible behaviors commonly observed in children with the disorder.

## Author Summary

Autism spectrum disorder (ASD) is a neurodevelopmental disorder associated with altered patterns of functional brain connectivity. Little is currently known about how moment-to-moment brain dynamics differ in children with ASD and typically developing (TD) children. Altered functional integrity of the midcingulo-insular network (M-CIN) has been implicated in the neurobiology of ASD. Here we use a novel coactivation analysis approach applied to a large sample of resting-state fMRI data collected from children with ASD and TD children to demonstrate altered patterns of M-CIN dynamics in children with the disorder. We speculate that these atypical patterns of brain dynamics may underlie behavioral inflexibility in ASD.

## INTRODUCTION

Autism spectrum disorders (ASDs) are neurodevelopmental disorders with central features of atypical social communication and restricted and repetitive behavioral patterns (American Psychiatric Association, 2013). These disorders have a high prevalence among school-aged children and adolescents in the United States, and have recently been estimated to affect around 1 in 40 individuals between the ages of 3 and 17 (Kogan et al., 2018). Because of the broad range and individual variability of the associated symptoms and etiologies of these disorders, they are difficult to understand and to treat. However, neuroimaging research in the past decade has identified brain network functional connectivity (FC) as a metric with which to potentially develop diagnostic indicators for ASD (Uddin et al., 2017). These studies have leveraged advances in machine learning to provide initial evidence that FC of the salience/midcingulo-insular (M-CIN) network (Uddin, Yeo, & Spreng, 2019), comprising the bilateral anterior insula and anterior cingulate cortices (Uddin, 2015), can discriminate ASD from typical development (Anderson et al., 2011; Uddin et al., 2013).

Previous FC and activation studies of ASD have indicated that atypical functioning of the M-CIN, default mode/medial fronto-parietal (M-FPN), and central executive/lateral fronto-parietal (L-FPN) is associated with the disorder (Abbott et al., 2016; Padmanabhan et al., 2017; Di Martino et al., 2009; Green et al., 2016). Because these brain networks support social and emotional behavior and executive functions, it is possible that altered relationships between them may underlie the social communication deficits and inflexible behaviors associated with ASD. Within the broader M-CIN, the anterior insula is a key node that orchestrates switching between the M-FPN and L-FPN (Menon & Uddin, 2010) and is posited to be a specific locus of dysfunction in ASD (Uddin & Menon, 2009; Nomi, Molnar-Szakacs, & Uddin, 2019). In studies of neurotypical individuals, the anterior insula has been shown to exhibit patterns of dynamic FC that link it transiently with the M-FPN (Nomi et al., 2016; Chang & Glover, 2010). Connectivity studies focusing on the anterior insula and the broader M-CIN in ASD have produced mixed findings, with some indicating that this network may be hyperconnected in children with the disorder (Uddin et al., 2013) while others report hypoconnectivity of this network (Abbott et al., 2016; Ebisch et al., 2011).

Traditional FC approaches operate under the assumption that the brain’s functional architecture remains relatively static throughout an entire fMRI scan. Although these static FC methods have provided informative estimates of the functional architecture of the brain, a growing body of evidence suggests that time-varying analysis of functional networks may reveal additional, dynamic aspects of brain function that have been previously overlooked (Calhoun et al., 2014; Hutchison et al., 2013). Because of this, dynamic or time-varying approaches have recently become an area of interest for characterizing brain function and dysfunction (Uddin & Karlsgodt, 2018; Lurie et al., 2020). In contrast to the single, average FC estimate produced by static approaches, dynamic analyses can identify multiple transient brain “states” or coactivation patterns (CAPs) that recur throughout an fMRI scan (Liu et al., 2018; Chen et al., 2015).

The current study uses a dynamic CAP approach based on previous research demonstrating that whole-brain patterns, captured at the peaks of a brain region’s signal, can resolve a traditional region of interest (ROI)–based FC map into multiple transient patterns occurring at different points in time. Liu and Duyn first showed that applying *k*-means clustering to spatial patterns derived from averaging across all brain voxels related to the highest ∼15% of activation time frames from the BOLD signal of a posterior cingulate cortex (PCC) ROI permits the identification of multiple different coactivation patterns, breaking down traditional static FC findings into multiple contributing patterns (Liu & Duyn, 2013). Chen and colleagues showed that a dynamic CAP analysis of a PCC node that employs *k*-means clustering across the highest activation time frames (instead of averaging across such time frames) produces dynamic CAPs that are less variable (i.e., more stable) than dynamic brain states identified using a sliding window correlation analysis (Chen et al., 2015). Additionally, unlike sliding window correlations, dynamic CAP analyses do not require the arbitrary selection of a window size over which to average FC measures (Allen et al., 2014). Thus, dynamic CAP analyses offer some advantages over sliding window dynamic FC approaches.

Compared with static FC methods, dynamic time-varying approaches may exhibit superior accuracy in characterizing the behavior of typically developing (TD) individuals, as well as in discriminating clinical from nonclinical populations including autism, schizophrenia, depression, and bipolar disorder (Chen et al., 2017; Rashid et al., 2018; Damaraju et al., 2014; Demirtas et al., 2016; Kaiser et al., 2019). Recent studies have used dynamic FC approaches to investigate ASD, revealing abnormal patterns of whole-brain network configurations (Uddin, 2020b). De Lacy et al. (2017) and Watanabe and Rees (2017) both reported a reduction in the number of transitions between brain state configurations in ASD, suggesting decreased functional flexibility or overly stable dynamic properties of the autistic brain. In both global and local brain networks, ASD has also been associated with reductions in other measures of dynamism, including functional fluidity and dynamic range (Fu et al., 2019). In a large sample of children, Rashid et al. (2018) found that those with autistic traits spent more time in a “globally disconnected” state. Similarly, Chen et al. (2017) found that individuals with ASD had generally weaker dynamic functional connections within whole-brain states. Individuals with ASD have also been shown to have longer mean dwell times in these whole-brain configurations with weaker connectivity (Yao et al., 2016). These studies provide evidence for consistent abnormalities in dynamic brain configurations in ASD, but have focused mostly on whole-brain network organization. In addition, no previous study has used a CAP analysis to probe brain dynamics in ASD. Further investigation is needed to determine whether the findings reported to date are related to specific brain networks implicated in ASD pathology such as the M-CIN (Uddin & Menon, 2009).

In the present study, we applied independent component analysis (ICA) to resting-state fMRI data collected from three cohorts of age- and IQ-matched children with ASD and TD children and conducted a dynamic CAP analysis focusing on the M-CIN. The goals of the study were to (a) identify patterns of dynamic CAPs related to the M-CIN, and (b) compare these dynamic M-CIN coactivation patterns between TD and ASD groups. We hypothesized that children with ASD would show altered M-CIN dynamics compared with TD children.

## METHODS AND MATERIALS

### Participants

**Figure 1.**
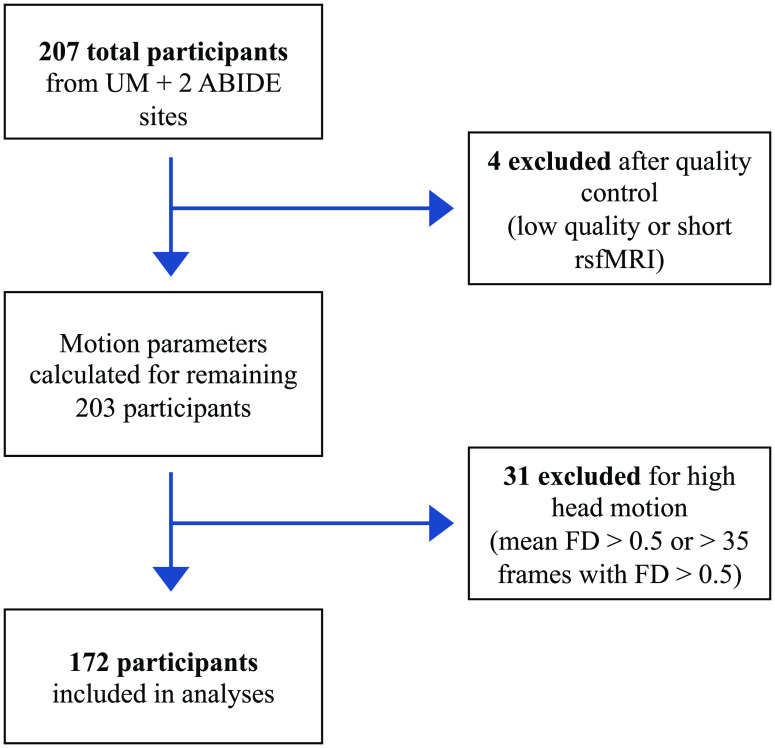
Participant selection process. Neuroimaging and phenotypic data from three sites (University of Miami, Erasmus University Medical Center Rotterdam, and Stanford University) were included.

Participant data were obtained from the Autism Brain Imaging Data Exchange (ABIDE I and II) public databases (Di Martino et al., 2014, 2017), combined with data collected by the Brain Connectivity and Cognition Laboratory at the University of Miami (UM). Institutional review board approval was provided by UM and each data contributor in the ABIDE databases. The sample selected from the databases met the following inclusion criteria: (a) subjects were between ages 6 and 13; (b) subjects had more than 160 volumes (5 minutes and 20 seconds) of resting-state fMRI data acquisition; (c) subjects had fMRI data collected using a repetition time (TR) = 2 seconds; and (d) subjects had their eyes closed during the resting-state scan (Nair et al., 2018). A total of 70 children scanned at UM met these criteria. Data for an additional 136 subjects were downloaded from the ABIDE databases. We selected the following sites that met these criteria: Stanford University (ABIDE I and II) and Erasmus University Medical Center Rotterdam (ABIDE II). After quality control and removal of subjects with high levels of head motion (mean framewise displacement (FD) > 0.5 mm and/or more than 35 frames with FD > 0.5 mm), the final sample consisted of 172 subjects (138 males) (Figure 1). The ASD (*n* = 75) and TD (*n* = 97) groups did not differ in terms of age (*p* = 0.285), full-scale IQ (*p* = 0.127), or mean head motion (*p* = 0.917). For participants from UM and Stanford University, ASD diagnosis was confirmed by a licensed psychologist using the Autism Diagnostic Observation Schedule (ADOS-2) (Lord et al., 2012). For the Erasmus Medical Center participants, an ASD diagnosis was confirmed from central medical records of the children’s general practitioners in the Netherlands. Additional demographic information for participants can be found in Table 1.

**Table 1.** Participant demographics

	**TD**	**ASD**	**P value**
Number of participants	97	75	–
Age (years)	9.79 ± 1.74 (6.3–13.2)	10.07 ± 1.70 (6.5–13.0)	0.285
Sex	74 males, 23 females	64 males, 11 females	–
Handedness	88 right, 4 left, 5 ambi	66 right, 6 left, 3 ambi	–
Full IQ	112.9 ± 13.9 (79–151)	108.8 ± 17.1 (67–141)	0.127
Verbal IQ	113.1 ± 15.7 (67–153)	108.3 ± 18.4 (72–149)	0.100
Performance IQ	107.5 ± 16.0 (71–152)	106.3 ± 15.5 (66–133)	0.632
Mean head motion (mm)	0.157 ± 0.088 (0.046–0.453)	0.155 ± 0.071 (0.055–0.430)	0.917

*Note*. Mean ± standard deviation (minimum-maximum). IQ was measured based on Wechsler abbreviated scale of intelligence (WASI, WASI-II) or Snijders–Oomen Nonverbal Intelligence Test (SON-R). TD, typically developing; ASD, autism spectrum disorder; IQ, intelligence quotient.

### MRI Data Acquisition

At all three data collection sites, participants underwent a mock scanning session to familiarize them with the MRI session procedure and offer them the opportunity to opt out before undergoing the MRI scan. For the resting-state fMRI scan, participants were instructed to keep their eyes closed while remaining awake. The scanner information and data acquisition parameters for each site are detailed in Supporting Information Table S1.

### Preprocessing of Resting-State fMRI Data

As the three sites utilized different scan lengths, each scan was trimmed to 155 timepoints, which was the length of the data collected at the Erasmus site. We removed the first five volumes from the beginning of the scans to remove any scanner initiation artifacts, and removed additional volumes from the end of the scans, as needed, to make each scan 5 minutes and 10 seconds in duration. The scans were then subjected to quality control procedures as follows. All neuroimaging data underwent a visual quality control procedure conducted by trained research assistants to identify scanner- or motion-induced artifacts. Artifacts included, but were not limited to, signal loss, head coverage, motion slicing, ringing, blurring, ghosting, and wrapping. Data were rated as either pass, qualified pass, or fail. All images included in the analysis received either a pass or a qualified pass rating. The resting-state scans were then preprocessed using DPABI version 3.1 (http://rfmri.org/dpabi) (Yan et al., 2016) and SPM version 12 (https://www.fil.ion.ucl.ac.uk/spm/software/spm12/). Preprocessing steps included realignment, normalization to the 3-mm MNI template, and smoothing (6 mm at full width at half maximum).

We did not apply global signal regression (GSR) in the current analysis following previous CAP studies (Chen et al., 2015; Kaiser et al., 2019). It is worth noting that in CAP analysis, functional networks are defined on the bases of instantaneous regional synchrony at each volume of data, and network definition does not rely on FC and thus may be less sensitive to the motion concerns inherent to FC analysis (Liu et al., 2018). The global signal has been demonstrated to contain both nuisance signals and neural signals (Murphy & Fox, 2017; Liu, Nalci, & Falahpour, 2017). Although removal of the global signal as a preprocessing step significantly mitigates artifacts from a variety of sources (Power et al., 2017; Ciric et al., 2018), it also results in removal of neuronal signal to a degree that is unknown in any given dataset (Uddin, 2017). Evidence from electrophysiological recordings in macaques (Scholvinck et al., 2010) and magnetic resonance spectroscopy studies in rodents (Hyder & Rothman, 2010) clearly demonstrates that the global signal also includes neural signals. The global signal has been further shown to have a direct neuronal source in studies of macaques (Turchi et al., 2018) and rats (Ma et al., 2020). In the current study, we were concerned that GSR might differentially affect the ASD and TD groups under investigation. As previously noted in simulation studies (Saad et al., 2012) and empirical studies of ASD, GSR can lead to a reversal in the direction of group correlation differences relative to other preprocessing approaches (Gotts et al., 2013). It is for these multiple reasons that we did not apply GSR in the current analysis, though we acknowledge that the costs and benefits associated with doing so depend on the research question and thus may differ from study to study (Uddin, 2020a).

### Group Independent Component Analysis

**Figure 2.**
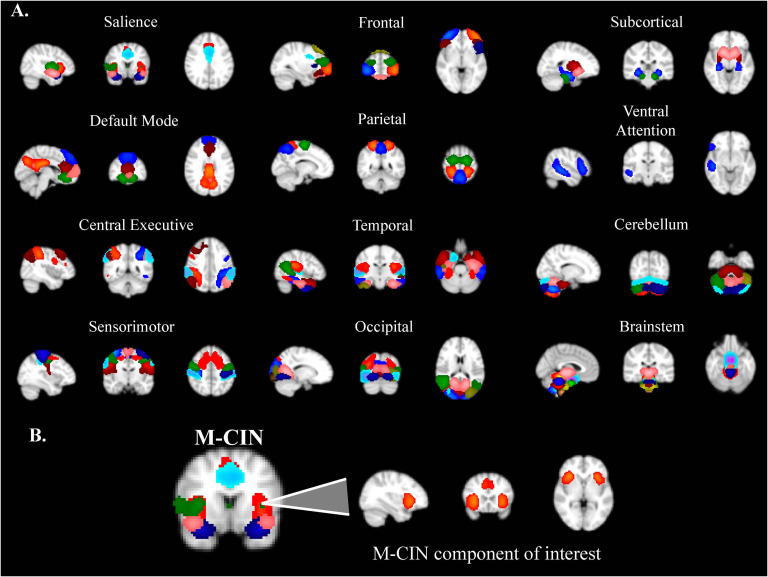
(A) Organization of ICA components into 12 functional networks. (B) Sagittal, coronal, and axial views of the salience/midcingulo-insular (M-CIN) component of interest in the analyses.

The resting-state fMRI data from all 172 participants were subjected to a high model order ICA by using the Group ICA of fMRI Toolbox (GIFT) (https://trendscenter.org/software/gift/). We used a model order of 100 independent components (ICs) as in previous similar work (Allen et al., 2014; Nomi et al., 2016), as individual brain networks are not always effectively separated when using lower model orders such as 25 or 50 (Ray et al., 2013), but can be too finely parcellated at higher model orders above 100 (Kiviniemi et al., 2009). The infomax algorithm was utilized for the ICA (http://mialab.mrn.org/software/gift) (Calhoun et al., 2001). To ensure the stability of this estimation, the ICA algorithm was repeated 20 times using ICASSO (http://www.cis.hut.fi/projects/ica/icasso). All 100 ICs were visually inspected and classified as noise or non-noise by two of the authors (JN & EM). The ICA components related to movement, white matter, or cerebrospinal fluid were removed from further analysis. The remaining 69 components were grouped into 12 functional domains to facilitate CAP interpretation: salience/M-CIN, default mode/M-FPN, central executive/L-FPN, sensorimotor, frontal, parietal, temporal, occipital, subcortical, attentional, cerebellum, and brainstem (Figure 2).

The network of interest in this study was the salience/M-CIN, with key nodes in the bilateral anterior insula and anterior cingulate cortices (Uddin, 2015), chosen because of its previous designation as a major locus of dysfunction in ASD (Uddin & Menon, 2009; Nomi, Molnar-Szakacs, & Uddin, 2019).

### Post-ICA Processing of fMRI Data

The time series were analyzed as a 155 (volumes) × 100 (ICs) matrix for each of the 172 participants, and were postprocessed using Matlab code from the GIFT toolbox. Postprocessing included linear detrending, despiking using the AFNI 3D despike command, and bandpass filtering (0.01–0.1 Hz) Allen et al. (2014).

### Statistical Analyses

**Figure 3.**
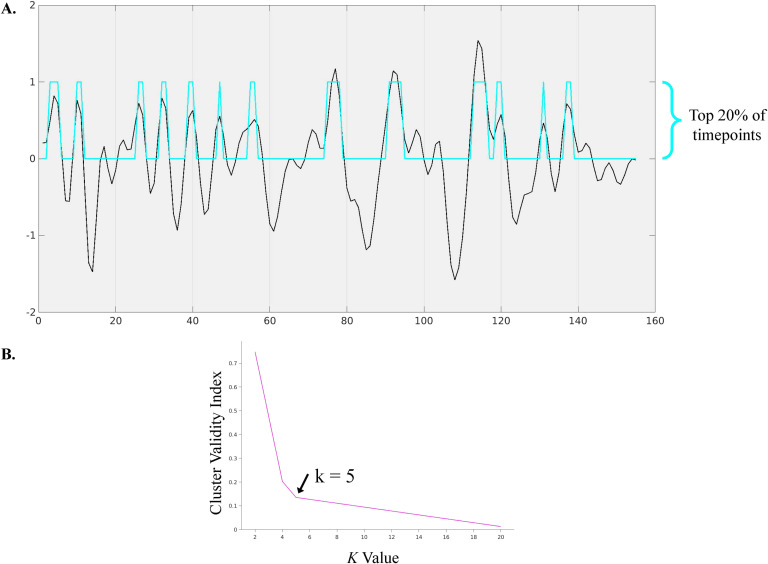
(A) Sample time course depicting extraction of top 20% of timepoints. Blue represents top 20% activation timepoints for salience/M-CIN component. (B) Elbow criteria graph to identify optimal *k* as 5.

A *k*-means clustering algorithm was applied to a concatenated matrix of all subjects’ timepoints of non-noise components to extract dynamic CAPs corresponding to different resting-state networks (Liu et al., 2018). For the anterior insula/anterior cingulate component we identified as the salience/M-CIN for each participant, timepoints with signal intensity among the top 20% (31 timepoints) and 30% (47 timepoints) of activation strength were extracted for analysis (Chen et al., 2015). This resulted in thresholding at the subject level. The optimal number of clusters was determined to be *k* = 5 by running *k*-means clustering with *k*-values 2 through 20 on a concatenated data matrix of all non-noise components for the selected timepoints across all participants. This optimal *k* was identified using the cluster validity index, defined as the elbow point (Figure 3) of a least-squares fit line plotted across the cluster validity index (the ratio between the within-cluster distance and the between-cluster distance) across all values of *k* (Allen et al., 2014). Following *k*-means clustering, the frequency of occurrence of each brain configuration was calculated for each participant and compared between groups.

Participants were divided into TD and ASD groups. For the top 20% and top 30% analyses, the mean frequencies of occurrence of CAPs were determined for the two groups. Frequency was computed as the proportion of occurrence for a specific brain state out of all possible brain states. Multiple linear regression models were used to compare the frequency of dynamic CAP occurrence between groups while controlling for data collection site (UM, Erasmus, or Stanford), mean head motion, age, and handedness.

Additional follow-up analyses were run to further explore patterns of activity when each group was clustered separately, to examine the relationship between head motion and CAPs, and to compare the current results against surrogate phase-randomized data. To determine if each group could be characterized by distinct CAPs rather than differences in frequency of the same CAPs, the ASD and TD groups were clustered separately using the optimal value of *k* as determined for all data. To determine if there was a relationship between specific CAPs, head motion and frequency of occurrence, average FD was computed for all TRs assigned to each CAP. Finally, to ensure that the identified CAPs were not driven by random differences in the data rather than brain activity, *k*-means clustering was conducted on phase-randomized surrogate data according to the procedure in Lancaster et al. (2018). The data were phase randomized producing surrogate data with the same mean, variance, and autocorrelation of the original time series. *K*-means clustering was then conducted using the optimal *k* value previously identified for the top 20% and top 30% of time points as in the previous analyses.

## RESULTS

**Figure 4.**
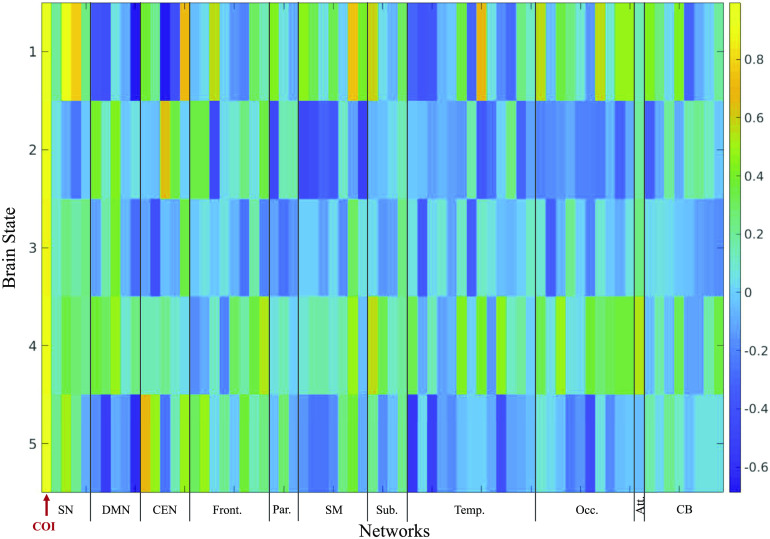
Activation of components within each brain network for Top 20% analysis. COI, component of interest; SN, salience network; DMN, default mode network; CEN, central executive network; Front., frontal; Par., parietal; SM, sensorimotor; Sub., subcortical; Temp., temporal; Occ., occipital; Att., attention; CB, cerebellum.

**Figure 5.**
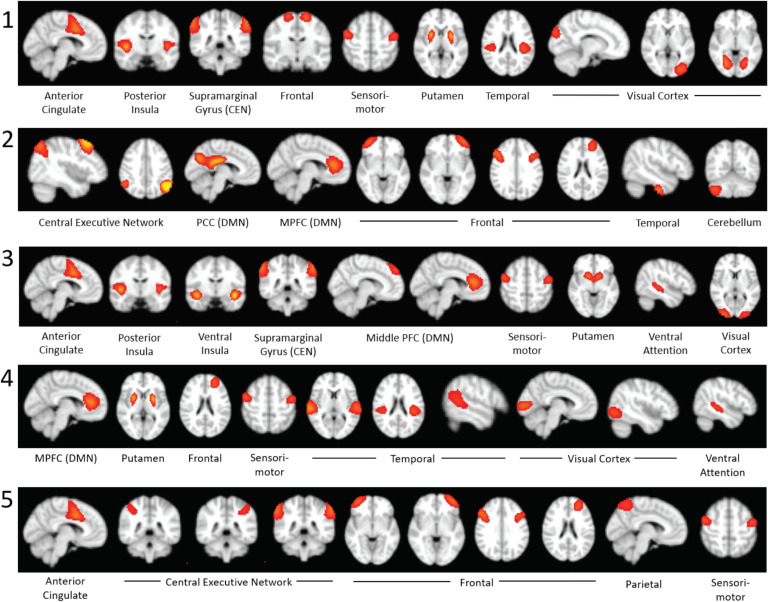
Top 10 most strongly activated components of each brain state in the top 20% analysis.

**Figure 6.**
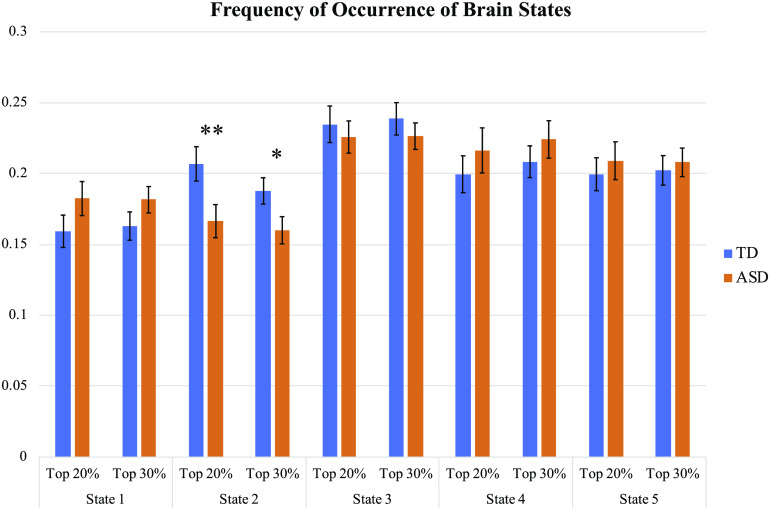
Observed frequency of occurrence of coactivation patterns 1–5 for typically developing (TD) and autism spectrum disorder (ASD) groups in the top 20% and top 30% analyses; *p < .05 in t test; **p < .05 in *t* test and multiple regression analyses.

For the optimal *k* of five clusters, the activation patterns for the top 20% of time points are shown in Figure 4. For each of the five clusters, the 10 components with the highest coactivation with the M-CIN component of interest were identified and are listed according to their brain network (Figure 5). The most notable brain configuration patterns were States 1 and 2. State 1 was characterized by strong coactivation with other components of the M-CIN. In both the top 20% and top 30% analyses, children with ASD entered this brain state more frequently than their TD counterparts, although this difference was not statistically significant (*p* = 0.1698, *p* = 0.1856) (Figure 6). State 2 was characterized by coactivation with the posterior cingulate cortex (PCC) and medial prefrontal cortex (mPFC) of the M-FPN, as well as bilateral regions of the L-FPN. This pattern of coactivation was significantly more frequently observed in the TD children compared with ASD in both analyses (*p* = 0.0198, *k* = 0.0232) (Figure 6). Significant group differences were not observed for frequency of occurrence of states 3–5. Results for the top 30% of time points are presented in Supporting Information Figures S1 and S2.

To ensure that our *k*-means clustering algorithm produced similar clusters in the ASD and TD participants, the above steps were run on the two groups, separately. There was no noticeable difference between the CAPs when the groups were clustered separately. The results of these analyses are detailed in Supporting Information Figures S3 and S4, and Supporting Information Table S2.

There was no identifiable systematic relationship between head motion, the frequency of occurrence, or amount of TRs in each CAP (Supporting Information Figure S5). The most frequently occurring CAP (e.g., the most TRs) did not have the highest average motion (CAP 4). The CAP with the lowest frequency of occurrence (CAP 1) did not have the average lowest motion (CAP 2). The distribution of TR FD for each CAP also did not appear to drive the clustering results (e.g., some CAPs with all TRs being high motion while other TRs were all low motion) while the average FD for each CAP was low (average FD < 0.2 mm).

Finally, phase-randomized surrogate data produced no noticeable CAPs of brain activity (Supporting Information Figure S6). The CAPs from the primary analysis show strong patterns of activation related to distinct brain networks. The CAPs from the phase-randomized surrogate data show weak activation across network nodes that does not appear to be related to distinct brain networks.

## DISCUSSION

ASDs are widely thought to be associated with atypical patterns of functional brain connectivity (Uddin, Supekar, & Menon, 2013; Vissers, Cohen, & Geurts, 2012; Müller et al., 2011) within and between large-scale brain networks important for high-level cognitive and emotional processes (Padmanabhan et al., 2017; Nomi, Molnar-Szakacs, & Uddin, 2019). Analysis of brain dynamics is beginning to reveal insights into neurodevelopmental disorders affecting brain connectivity (Uddin & Karlsgodt, 2018; White & Calhoun, 2019). This emerging literature provides initial evidence for alterations in ASD related to the number of transitions between brain states (Uddin, 2020a). Most of these studies have focused on characterizing whole-brain dynamic patterns and utilized sliding window dynamic FC approaches that have known potential pitfalls such as arbitrary window sizes (Preti, Bolton, & Van De Ville, 2017; Lurie et al., 2020). Here we focus specifically on dynamics of a brain network known as the salience network, or M-CIN, with key nodes in the bilateral anterior insula and anterior cingulate cortices (Uddin, 2015), thought to be a locus of dysfunction in ASD (Uddin & Menon, 2009; Nomi, Molnar-Szakacs, & Uddin, 2019). We use dynamic coactivation pattern (CAP) analysis, which relies on fewer assumptions than the sliding window approach, and permits the examination of brain state alterations closer to the temporal resolution of individual time frames (Chen et al., 2015). Using a combined ICA and CAP analytic approach, we investigated the dynamic nature of M-CIN organization in children with ASD. By utilizing only time points with the highest overall activation in the M-CIN in our analyses, we were able to focus on this key network and note differences in the frequency of its coactivation with other major large-scale networks of the brain.

In three of the five dynamic states or CAPs identified across TD and ASD participants, the component of interest was consistently coactivated with other components of the M-CIN, including the anterior cingulate and the posterior and ventral regions of the insular cortex. This coactivation was particularly strong in State 1 and was also observed to some extent in States 3 and 4, which showed similar coactivation of the M-CIN components. Previous static FC work has demonstrated altered M-CIN network properties in children and adolescents with ASD (Ebisch et al., 2011; Abbott et al., 2016), and FC of the M-CIN can be used to discriminate ASD from typical development (Uddin et al., 2013; Anderson et al., 2011).

The relative frequency of occurrence of State 2 differed significantly between the TD and ASD groups. In this state, the component of interest was not strongly coactivated with any other components of the M-CIN, and its coactivation with components of other networks was relatively weak, except a single component in the central executive/lateral fronto-parietal network (L-FPN) and two components in the default mode/medial fronto-parietal network (M-FPN). Children with ASD exhibited this CAP significantly less frequently than TD children. The lower frequency of occurrence of this state for children with ASD indicates less coactivation of the M-CIN with components of the M-FPN and L-FPN, especially the medial prefrontal cortex (mPFC) and PCC. Previous work has demonstrated that effective connectivity among nodes of these three networks can be used to discriminate task-evoked and resting states in TD children to a greater extent than in children with ASD. The same study found that this brain state discriminability was related to symptom severity in the domain of restricted and repetitive behaviors in children with ASD (Uddin et al., 2015). The reduced communication among these three networks observed in our ASD samples could be related to symptoms of cognitive and behavioral inflexibility commonly observed in children with the disorder.

With regard to the L-FPN and M-FPN, two other major networks that have been implicated in autism and other neurodevelopmental disorders, components of these networks were consistently among the 10 with strongest coactivation with the M-CIN in all 5 observed CAPs. Within the M-FPN, the components with high activation included the mPFC in States 2, 3, and 4 as well as the PCC in State 2. For the L-FPN, the supramarginal gyrus (SMG) was a top 10 component in States 1 and 3, and bilateral regions of the L-FPN were strongly activated in States 2 and 5. As activation of the L-FPN and M-FPN has been shown to be influenced and controlled by the M-CIN (Uddin, 2015), this commonly observed CAP is consistent with previous research examining interactions among these three networks (Goulden et al., 2014).

Only one previous study has specifically examined anterior insula dynamics in ASD. Using a large ABIDE sample, Guo and colleagues identified an anterior insula ROI using Neurosynth (Yarkoni et al., 2011); they then used a sliding window analytic approach to show that this ROI exhibited decreased FC with the mPFC and PCC in certain brain states compared with TD individuals (Guo et al., 2019). Our current findings of reduced coactivation between M-CIN and M-FPN in ASD is in line with this observation, and employs a method that overcomes some of the limitations of sliding window correlation analyses (Lurie et al., 2020; Preti, Bolton, & Van De Ville, 2017).

### Limitations

A few limitations of the current work should be noted. First, our sample includes data collected at three different sites. While this increases the sample size and statistical power, the use of data across multiple sites presents its own limitations in that intersite variability may affect the analyses. Though we selected data from a limited age range by using similar acquisition parameters and participant eye status (eyes closed), and attempted to control for acquisition site across all analyses, we cannot be sure that inherent between-site effects are completely accounted for. In addition, we cannot ascertain whether diagnostic procedures and scanner sessions were conducted in the same way for all participants at the three sites. There were also some discrepancies in the information that was reported to the ABIDE database for the different sites, eliminating the possibility of using behavioral measures like the Repetitive Behavior Scale or the Social Communication Questionnaire to explore relationships between dynamic FC parameters and symptom severity in the ASD group. Furthermore, similarly to most studies of ASD, our sample consisted mostly of males, with only 34 female participants included in the analyses. Although sex was included as a variable in our statistical analyses and had minimal effect on the significant results reported, this imbalance of males to females may fail to account for differences in the brain activity of the two sexes (Lai et al., 2017).

Despite these limitations, this study identifies the M-CIN, including the bilateral anterior insula and anterior cingulate, as a network of particular interest for further investigation with dynamic FC approaches in children with ASD. Further research could use tasks known to elicit activation in this network to investigate the differential activation in children and adolescents with ASD (Odriozola et al., 2016), as well as adults and older individuals, as patterns of dynamic FC may change significantly with age (Hutchison & Morton, 2015, 2016). The relationship between the M-CIN, M-FPN, and L-FPN in ASD also merits further investigation, as the identification of connections or dynamic coactivations (or lack thereof) between these networks may be utilized in the future as potential diagnostic identifiers of ASD and other neurodevelopmental disorders (Uddin et al., 2017).

### Conclusion

The findings of this study build on the growing body of literature on the use of dynamic connectivity approaches in the investigation of neurodevelopmental disorders, and ASD in particular. Our combination of ICA and CAP allowed for the identification of highly specific networks of interest, particularly those centered on the bilateral insular and anterior cingulate cortices. The results of this study provide further insight into the dynamic FC abnormalities that may underlie the clinical presentation of ASD, but future studies are needed to identify the neural mechanisms by which these brain abnormalities are related to ASD symptoms, particularly those related to inflexible behaviors.

## ACKNOWLEDGMENTS

The authors gratefully acknowledge Willa Voorhies, Paola Odriozola, Kristafor Farrant, Dina Dajani, Casey Burrows, Taylor Bolt and Shruti Vij for assistance with MRI data collection, as well as Amy Beaumont, Sandra Cardona, Meaghan Parlade, and Michael Alessandri for assistance with clinical assessments.

## SUPPORTING INFORMATION

Supporting information for this article is available at https://www.doi.org/10.1162/netn_a_00163.

## AUTHOR CONTRIBUTIONS

Emily Marshall: Data curation; Formal analysis; Visualization; Writing - Original Draft. Jason Nomi: Conceptualization; Formal analysis; Funding acquisition; Investigation; Supervision; Visualization; Writing - Original Draft; Writing - Review & Editing. Bryce Dirks: Data curation; Supervision; Writing - Review & Editing. Celia Romero: Conceptualization; Data curation; Supervision; Writing - Review & Editing. Lauren Kupis: Formal analysis; Supervision; Writing - Review & Editing. Catie Chang: Formal analysis; Methodology; Resources; Writing - Review & Editing. Lucina Q. Uddin: Conceptualization; Funding acquisition; Project administration; Resources; Supervision; Writing - Original Draft; Writing - Review & Editing.

## FUNDING INFORMATION

Lucina Q. Uddin, National Institute of Mental Health (http://dx.doi.org/10.13039/100000025), Award ID: R01MH107549. Lucina Q. Uddin, Canadian Institute for Advanced Research. Lucina Q. Uddin, University of Miami Gabelli Senior Scholar Award. Jason Nomi, National Institute of Mental Health (http://dx.doi.org/10.13039/100000025), Award ID: R03MH121668. Jason Nomi, Brain & Behavior Research Foundation.

## TECHNICAL TERMS

Midcingulo-insular network: Core cortical brain regions within the midcingulo-insular network (M-CIN) include bilateral anterior insula and anterior midcingulate cortex. This network encapsulates the previously characterized “salience,” “ventral attention,” and “cingulo- opercular” networks.
Medial fronto-parietal network: Core cortical brain regions within the medial fronto-parietal network (M-FPN) include medial prefrontal and posterior cingulate cortex. This network is also referred to as the “default mode” network.
Lateral fronto-parietal network: Core cortical brain regions within the lateral fronto-parietal network (L-FPN) include lateral prefrontal and anterior inferior parietal cortex. This network is also referred to as the “control” or “central executive” network.
Coactivation pattern analysis: Coactivation pattern (CAP) analysis tracks brain state alterations at the individual time frame level, and is one approach for quantifying brain dynamics.
Global signal regression: Global signal regression (GSR) refers to the removal of the average value of all whole-brain or gray matter signals via regression as a resting state fMRI data denoising strategy.
