# Supplementary material for: Coactivation pattern analysis reveals altered salience network dynamics in children with autism spectrum disorder
Source: Netw Neurosci. 2020 Dec 1;4(4):1219–34. doi: 10.1162/netn_a_00163 (PMC7781614; doi:10.1162/netn_a_00163)
Supplement: Supplementary file 1 [file netn-04-1219-s001.pdf]

Marshall, E., Nomi, J. S., Dirks, B., Romero, C., Kupis, L., Chang, C. & Uddin, L. Q. (2020). Supporting information for “Co-activation pattern analysis reveals altered salience network dynamics in children with autism spectrum disorder.” *Network Neuroscience*, *4*(4), 1219–1234.
https://doi.org/10.1162/netn_a_00163

Supplementary Information

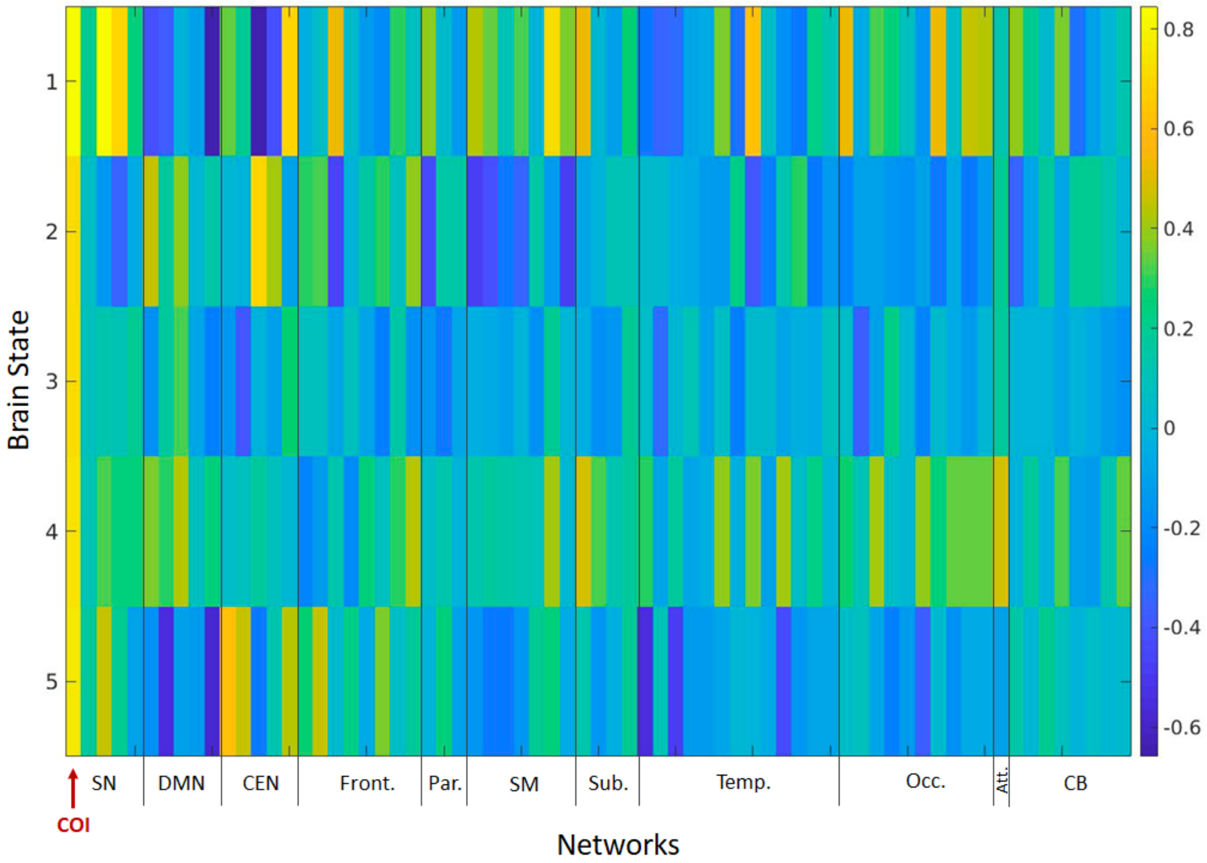

Supplementary Figure 1. Activation of components of each brain network for Top 30% analysis. COI = component of interest (M-CIN).

Marshall, E., Nomi, J. S., Dirks, B., Romero, C., Kupis, L., Chang, C. & Uddin, L. Q. (2020). Supporting information for "Co-activation pattern analysis reveals altered salience network dynamics in children with autism spectrum disorder." *Network Neuroscience*. Advance publication.
https://doi.org/10.1162/netn_a_00163

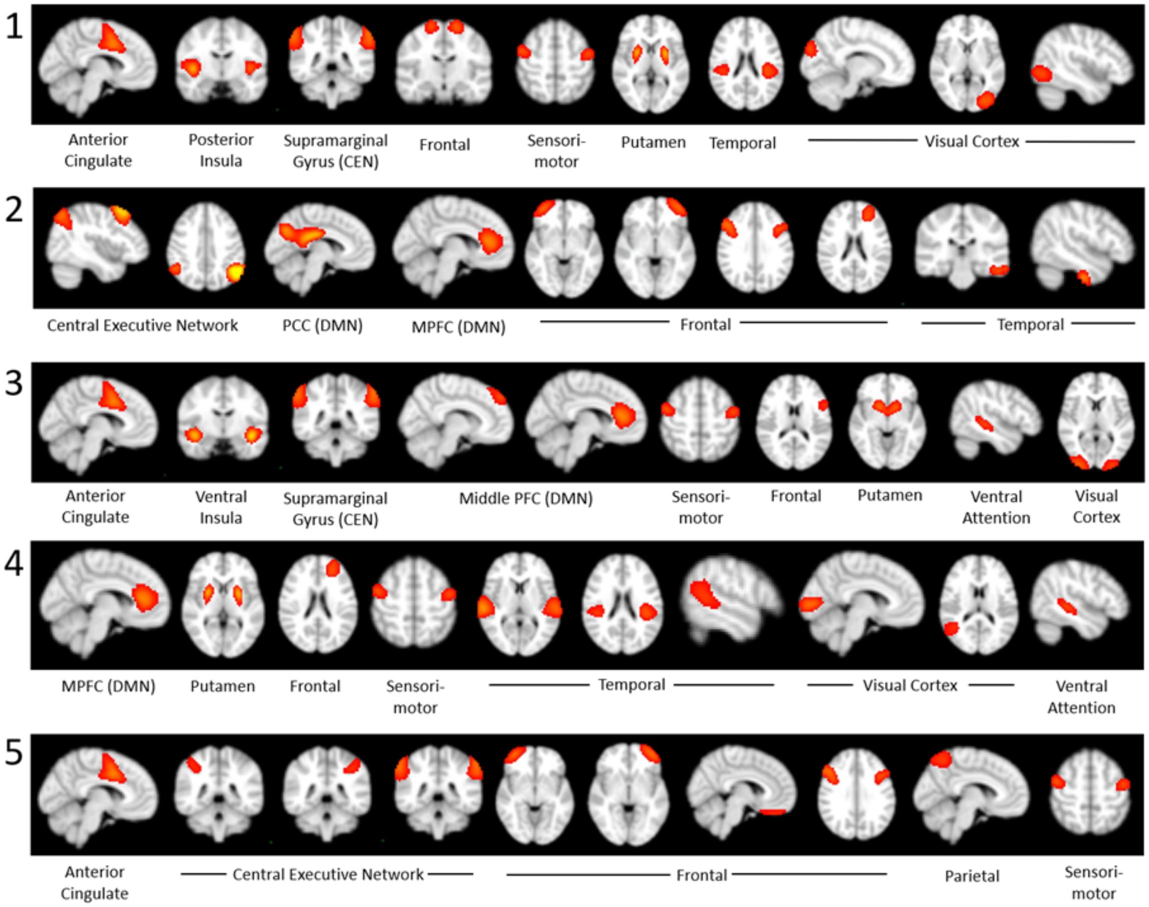

Supplementary Figure 2. Top 10 most strongly activated components of each brain state in the Top 30% analysis.

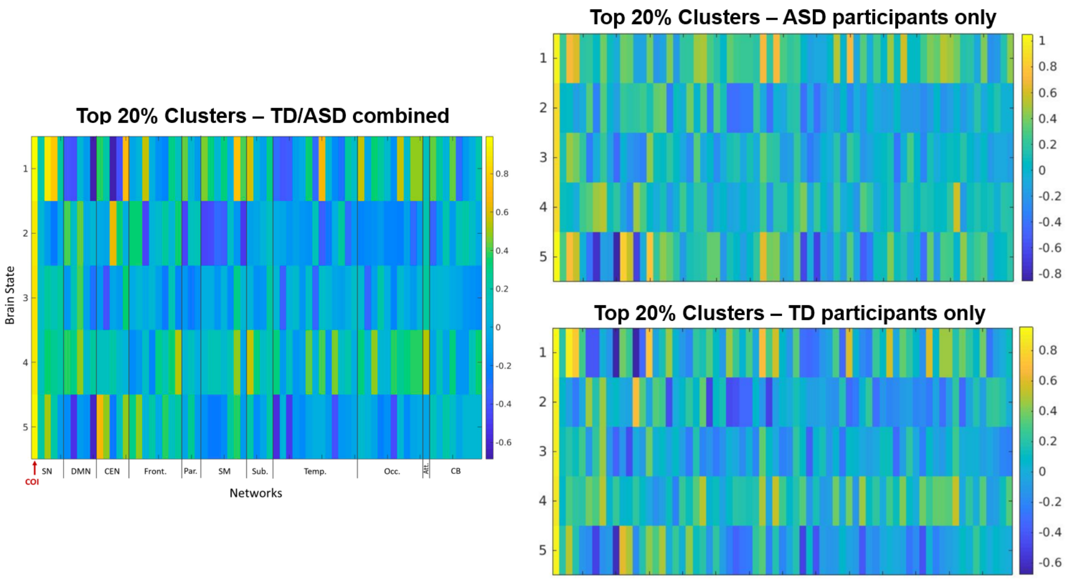

Supplementary Figure 3. Left: Top 20% activation clusters for TD and ASD participants, combined; Right: Top 20% activation clusters for TD and ASD participants, separately.

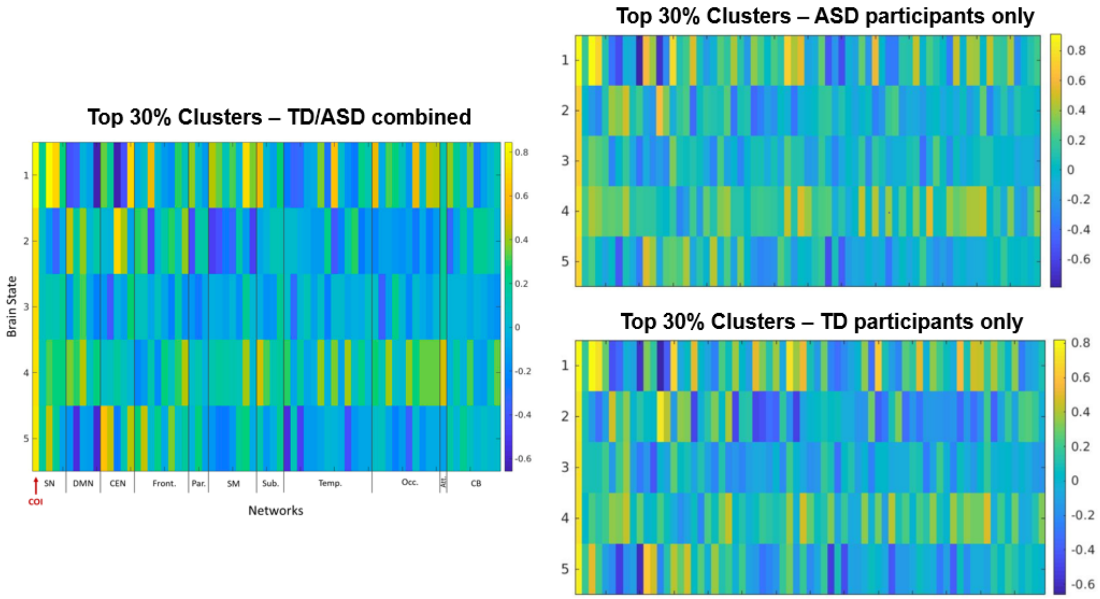

Supplementary Figure 4. Left: Top 30% activation clusters for TD and ASD participants, combined; Right: Top 30% activation clusters for TD and ASD participants, separately.

Marshall, E., Nomi, J. S., Dirks, B., Romero, C., Kupis, L., Chang, C. & Uddin, L. Q. (2020). Supporting information for “Co-activation pattern analysis reveals altered salience network dynamics in children with autism spectrum disorder.” *Network Neuroscience*. Advance publication. https://doi.org/10.1162/netn_a_00163

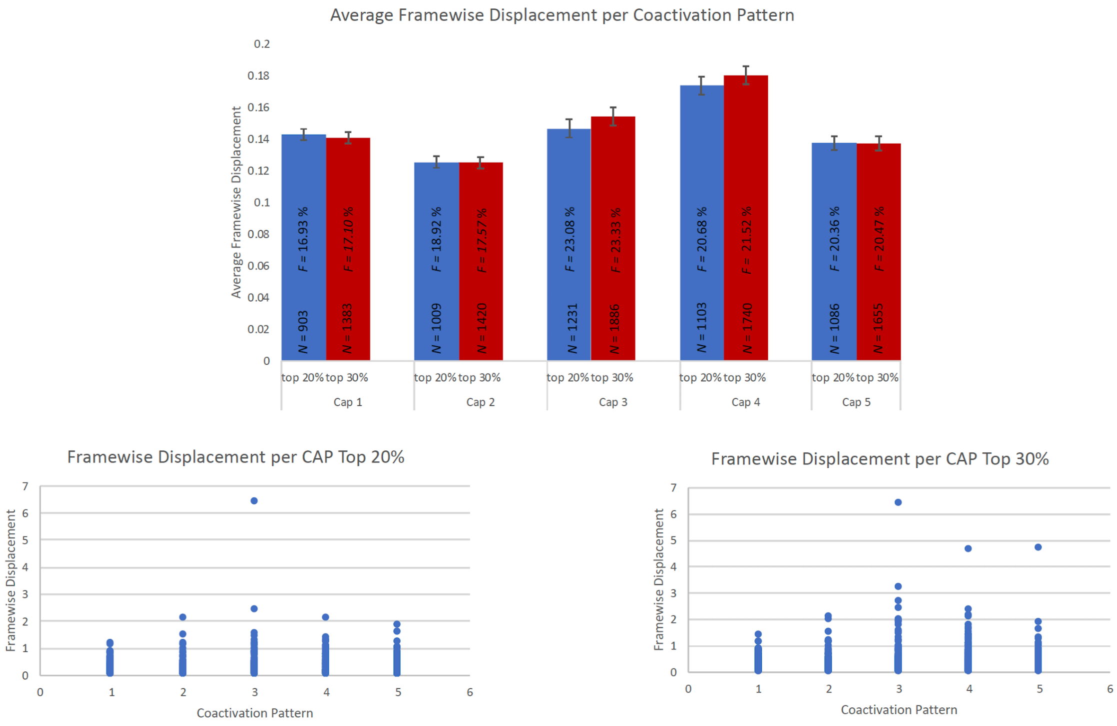

Supplementary Figure 5. Top: average FD across TRs for each CAP in the top 20% and top 30% analyses. Bottom: distribution of FD for each TR across all CAPs for the top 20% and top 30% analyses. F = frequency of occurrence for each CAP. N = number of TRs within each CAP.

Marshall, E., Nomi, J. S., Dirks, B., Romero, C., Kupis, L., Chang, C. & Uddin, L. Q. (2020). Supporting information for “Co-activation pattern analysis reveals altered salience network dynamics in children with autism spectrum disorder.” *Network Neuroscience*. Advance publication.
https://doi.org/10.1162/netn_a_00163

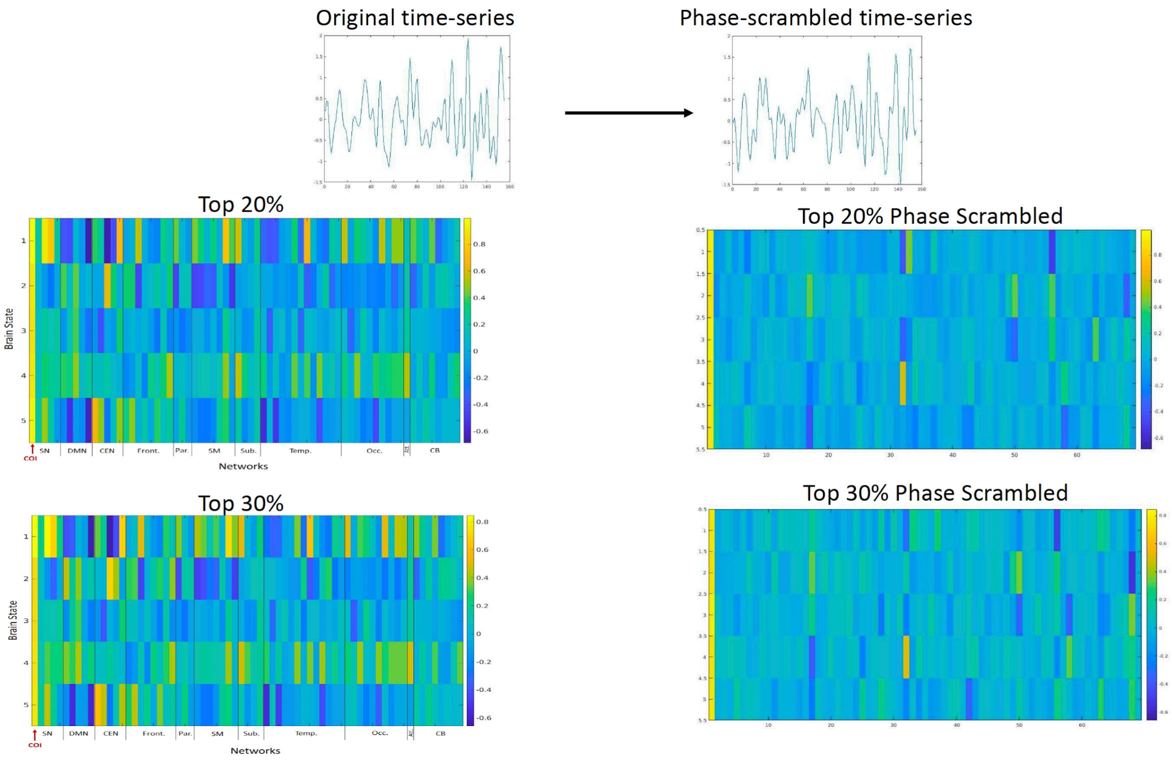

Supplementary Figure 6. Phase randomized surrogate data were created from the original subject data resulting in time-series with the same mean, variance, and auto-correlation as the original time-series. Clustering on the phase scrambled data does not produce distinct network activation patterns that can be found in the original analysis on the empirical data.

Supplementary Table 1. Data acquisition details for three ABIDE sites included in analyses.

	**University of Miami**	**Stanford University**	**Erasmus University Medical Center Rotterdam**
**Scanner**	3T General Electric Healthcare	3T General Electric Signa	3T General Electric Discovery MR750
**Headcoil**	32-channel	48-channel	8-channel
**Sequence**	EPI	EPI	EPI
**Acquisition Time [min:sec]**	10:00	6:00	5:20
**Repetition Time (TR) [ms]**	2000	2000	2000
**Echo Time (TE) [ms]**	30	30	30
**Number, Orientation & Thickness of Slices**	42, Axial, 3.4 mm	29, Axial, 4.5 mm (ABIDE I)<br>31, Axial, 4 mm (ABIDE II)	37, Axial, 4 mm
**Field of view [mm]**	220	200 (ABIDE I)<br>220 (ABIDE II)	230
**Matrix size**	64 x 64	64 x 64	64 x 64
**Flip Angle [degrees]**	75	80	85

Supplementary Table 2. Top 20% (top) and 30% (bottom) average frequencies of occurrence of clusters for TD participants only and ASD participants only, compared with averages for TD and ASD groups combined.

TD

Cluster	Initial Average	TD Subjects Only
1	0.1593	0.1869
2	0.2069	0.1935
3	0.2348	0.2185
4	0.1995	0.2035
5	0.1995	0.1975

ASD

Cluster	Initial Average	ASD Subjects Only
1	0.1824	0.1991
2	0.1665	0.2099
3	0.2258	0.2348
4	0.2163	0.2030
5	0.2090	0.2146

TD

Cluster	Initial Average	TD Subjects Only
1	0.1630	0.1707
2	0.1878	0.1685
3	0.2386	0.2378
4	0.2084	0.2161
5	0.2022	0.2071

ASD

Cluster	Initial Average	ASD Subjects Only
1	0.1816	0.1543
2	0.16	0.1915
3	0.2264	0.2346
4	0.2241	0.2142
5	0.2079	0.2054

Marshall, E., Nomi, J. S., Dirks, B., Romero, C., Kupis, L., Chang, C. & Uddin, L. Q. (2020). Supporting information for "Co-activation pattern analysis reveals altered salience network dynamics in children with autism spectrum disorder." *Network Neuroscience*. Advance publication.
https://doi.org/10.1162/netn_a_00163
